# Integrative Approaches to Sleep Management in Skin Disease: Systematic Review

**DOI:** 10.2196/48713

**Published:** 2023-12-13

**Authors:** Vishnutheertha A Kulkarni, Isaiah Mojica, Vahram Gamsarian, Michelle Tahjian, David Liu, Tjinder Grewal, Yuyang Liu, Torunn E Sivesind, Peter Lio

**Affiliations:** 1 University of Queensland Medical School Brisbane Australia; 2 University of Cincinnati College of Medicine Cincinnati, OH United States; 3 University of Michigan School of Medicine Ann Arbor, MI United States; 4 Nova Southeastern University Dr Kiran C Patel College of Osteopathic Medicine Fort Lauderdale, FL United States; 5 University of Toledo College of Medicine Toledo, OH United States; 6 University of Colorado Anschutz Medical Campus Aurora, CO United States; 7 Northwestern University Feinberg School of Medicine Chicago, IL United States

**Keywords:** sleep, dermatology, atopic dermatitis, chronic idiopathic urticaria, quality of life, literature review, parameter, teledermatology, dermatologist, skin, epidermis, review, polysomnography, polysomnographic, sleep medicine

## Abstract

**Background:**

Dermatological conditions, especially when severe, can lead to sleep disturbances that affect a patient’s quality of life. However, limited research exists on the efficacy of treatments for improving sleep parameters in skin conditions.

**Objective:**

The objective was to perform a systematic review of the literature on dermatological conditions and the treatments available for improving sleep parameters.

**Methods:**

A literature review was performed using the PubMed, Ovid MEDLINE, Embase, Cochrane, and ClinicalTrials.gov databases from 1945 to 2021. After filtering based on our exclusion criteria, studies were graded using the SORT (Strength of Recommendation Taxonomy) algorithm, and only those receiving a grade of “2” or better were included.

**Results:**

In total, 25 treatment studies (n=11,025) assessing sleep parameters related to dermatological conditions were found. Dupilumab appeared to be the best-supported and most effective treatment for improving sleep in atopic dermatitis (AD) but had frequent adverse effects. Topical treatments for AD were mostly ineffective, but procedural treatments showed some promise. Treatments for other conditions appeared efficacious.

**Conclusions:**

The evaluation of sleep parameter changes in dermatological treatments is predominantly restricted to AD. Systemic interventions such as dupilumab and procedural interventions were the most efficacious. Sleep changes in other dermatoses were limited by a paucity of available studies. The inclusion of a sleep assessment component to a broader range of dermatological treatment studies is warranted.

## Introduction

The importance of sleep and the consequences of sleep deprivation on the patient’s quality of life have been thoroughly defined, with decreased health-related quality of life survey measures and daytime impairment [1,2]. Dermatological conditions can affect patients’ lives in numerous ways, with sleep disturbance as one of the most debilitating effects. Sleep may cause aberrations of skin functions, specifically with thermoregulation and fluid balance maintenance. Disruptions in these regulatory mechanisms may contribute to nocturnal pruritus [3,4].

The impact of these conditions on sleep, whether in terms of quality or duration, however, remains understudied. The efficacy of different dermatological treatments for improving sleep parameters is not always clear to clinicians. In this review, we performed a literature search regarding the effects of different treatments on sleep disturbances across dermatological conditions. After filtering studies according to our exclusion criteria, those relating to atopic dermatitis, pruritus and xerosis, prurigo nodularis, and chronic idiopathic urticaria were found.

## Methods

**Figure 1:**
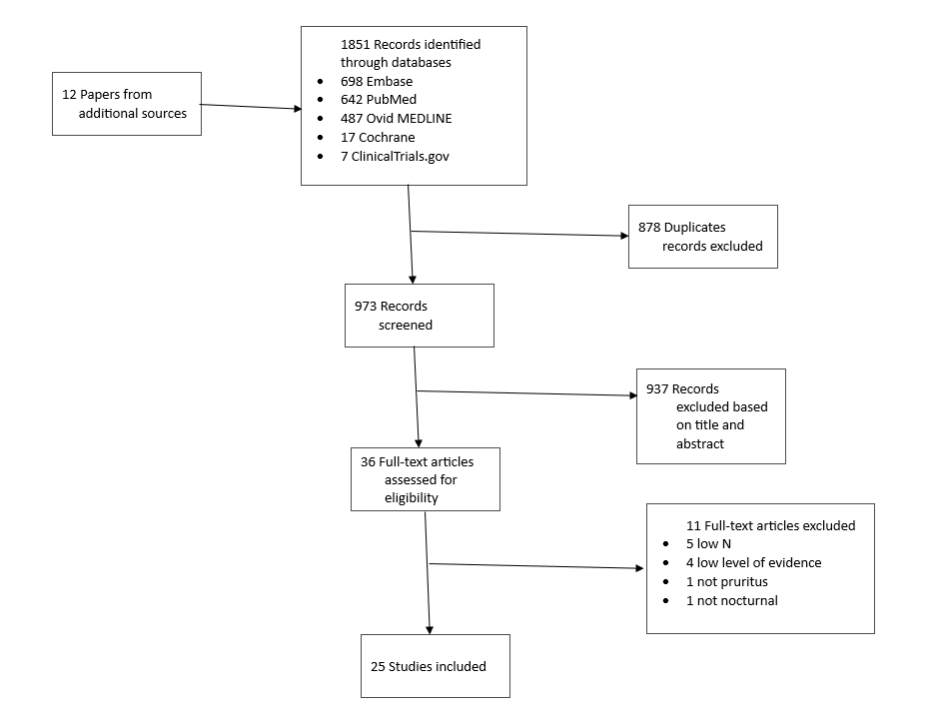
PRISMA (Preferred Reporting Items for Systematic Reviews and Meta-Analyses) flowchart.

We conducted a literature search using the keywords “sleep disturbance,” “dermatology,” and “management” from the PubMed, Ovid MEDLINE, Embase, Cochrane, and ClinicalTrials.gov databases. We included studies from 1945 to September 2021 in our initial review, yielding 1863 results. After discarding duplicates, 973 unique studies were analyzed by 2 independent reviewers (VG and IM) for relevant information pertaining to treatments for sleep management in dermatological conditions, yielding 25 studies for further analysis (Figure 1 and Multimedia Appendix 1 [5]).

All disputes were settled by a third independent reviewer (VK). Inclusion criteria included peer-reviewed original articles involving various treatment modalities for sleep management in the form of randomized controlled trials (RCTs) and cohort studies. Only studies that focused solely on dermatological conditions were included. The SORT (Strength of Recommendation Taxonomy) algorithm was used to quantify the level of evidence [6]. Studies that received a level of evidence score worse than a “2” were excluded.

## Results

### Characteristics of Included Studies

The 26 studies included in this review encompass a total of 11,022 patients. Of these, 21 studies were included for AD, encompassing 4111 patients (n=164, ~4% pediatric patients). A total of 2 studies were included for pruritus and xerosis, including 5965 patients. Only 1 study was included for prurigo nodularis, psoriasis vulgaris, and chronic idiopathic pruritus, analyzing 27 patients, 394 patients, and 525 patients, respectively. Collectively, the studies spanned a period from 1979 to 2021.

### Treatment for Atopic Dermatitis

#### Overview

A wide array of studies has evaluated the efficacy of treatments for AD and concomitant sleep disturbances. These can broadly be arranged into 3 groups, according to whether the treatment modality was systemic, topical, or procedural. Each of the study details, with respective treatment adverse effects, is further delineated in Tables 1 and 2. Sources of funding for each study are provided in Table 3.

**Table 1:** Treatments for sleep management in skin disease.

Study	Disease	Intervention	Level of evidence	Study design	Sample size, N	Dosage	Sleep scale	Outcomes
Fargnoli et al [7] (2019)	AD^a^	Dupilumab	2	Retrospective cohort	109	Single subcutaneous injection of 600 mg, then 300 mg Q2W^b^	Sleep-NRS^c^	Reduction from 6.9 (SD 2.5) to 3.3 (SD 2.4) at 4 wk and to 1.9 (SD 2.2) at 16 wk (*P*<.001 for both)
Cork et al [8] (2019)	AD	Dupilumab	1	Pooled data from RCTs^d^	1379	Subcutaneous injection of 300 mg QW^e^ Subcutaneous injections of 300 mg Q2W	SCORAD^f^ POEM^g^	Reduction by 3.4 (QW) and 3.3 (Q2W) vs 0.82 (placebo) (*P*<.001) More patients in the text group reported the absence of sleep disturbance: 51.2% (n=234, Q2W), 43.5% (n=199, QW), 17.6%, (placebo, n=81; *P*<.001)
Tsianakas et al [9] (2017)	AD	Dupilumab	1	RCT	64	300 mg QW subcutaneous dupilumab or placebo for 12 wk	SCORAD VAS^h^ scores for pruritus and sleep loss	Decrease in mean % change from baseline when compared to placebo of −65.6 (17.78, 95% CI 101.27-30.01; *P*<.001)
Tofte et al [10] (2018)	AD	Dupilumab	1	Pooled data from RCTs	233	300 mg QW and 300 mg Q2W vs placebo	SCORAD VAS for sleep loss	Decrease in SCORAD VAS sleep loss score at wk 12: QW: 75.4% (5.13, n=118) vs 19.7%, (6.17, n=115) for placebo (*P*=.0001); Q2W: 51.9% (10.79, n=64) vs 3.8%, (11.55, n=61) for placebo (*P*=.001)
Paller [11] (ongoing clinical trial)	AD	Dupilumab	2	Open label	40 (pediatric)	Weight-based dosage for 12 wk	PROMIS^i^ Wake after sleep onset	None posted
Chang et al [12] (2016)	AD	Melatonin	1	RCT	73 (pediatric)	3 mg QD^j^ for 4 wk	SCORAD Actigraphy Subjective description	Reduction by 9.1 (95% CI –13.7 to –4.6; *P*<.001) Reduction of the sleep-onset latency by 21.4 min compared with placebo^k^ (95% CI –38.6 to –4.2; *P*=.02) No significant difference in subjective measures
Harper et al [13] (2000)	AD	Cyclosporine	2	Prospective, parallel, open label	40 (pediatric)	Maximum 5 mg/kg QD	SASSAD^l^ score and area of involvement (rule of 9s)	Significant improvement was seen in all treatment groups. (no *P* values given)
Silverberg et al [14] (2021)	AD	Abrocitinib	1	Pooled data from RCTs	942	Oral 200 mg, 100-mg monotherapy, or placebo QD for 12 wk	NTIS^m^	≥4-point improvement in NTIS; 57% (n=207), 42.7% (n=158), and 12.7% (n=27); no *P* values given Difference in proportion of patients who achieved NTIS response at wk 12 vs placebo 200 mg: 44.6% (n=162; *P*<.001) 100 mg: 29.8% (n=110; *P*<.001)
Lio et al [15] (2021)	AD	Baricitinib	1	Prospective analysis from RCT data	440	1 mg or 2 mg QD for 16 wk	ADSS^n^	For patients with BSA^o^ involvement of 10%-50%, a decrease of at least 1.5 points in number of nighttime awakenings caused by itch was observed for 2 mg (*P*=.001) but not 1 mg (*P*=.15) vs placebo For patients with baseline ADSS Item 2 score ≥1.5, a decrease of at least 1.5 points in number of nighttime awakenings caused by itch was observed for 1 mg (*P*=.04) and 2 mg (*P*<.001) vs placebo
Kawana et al [16] (2010)	AD	Tandospirone citrate	1	RCT	37	30 mg QD for 4 wk	SCORAD index and VAS for insomnia	There was a significant decrease in the SCORAD Index after 4 wk for the TC^p^ group (before treatment 49.6, after treatment 36.4, change –13.2; *P*<.001), but not in the untreated group (before treatment 44.5, after treatment 37.9, change –6.7 No significant difference in SCORAD or VAS insomnia scores between groups
Munday et al [17] (2002)	AD	Chlorpheniramine	1	RCT	151	2.5 mL (1-5 y) or 5 mL (6-12 y) QDpm^q^	The severity of daytime drowsiness, number of episodes of sleeplessness due to scratching	No significant difference when compared to placebo
Ebata et al [18] (1997)	AD	Nitrazepam	2	RCT crossover	10	5 mg QD for 2 nights, then 5 mg BID^r^ for 1 night, followed by a 4-d washout period	TST^s^ divided by total recording time (TST%)	Decrease in frequency of scratching bouts but increase in mean duration. Thus, no significant difference in TST%
Savin et al [19] (1979)	AD	Trimeprazine and trimipramine	2	RCT	12	20 mg trimeprazine tartrate, 50 mg trimipramine maleate, or placebo, taken 1 h before sleeping for 3 nights	EEG^t^ recordings: 20-s periods in terms of wakefulness and the usual stages of sleep (1, 2, and 3 or 4)	No significant differences were found among the 3 treatments for total time asleep, time spent before falling asleep, or in total wakefulness after first falling asleep. There was a tendency toward longer sleep duration and shorter sleep latency with both drugs
Parikh-Das et al [20] (2017)	AD	Colloidal oatmeal cream	1	RCT	23 (pediatric)	Applied topically for 14 d “per the label instructions”	Clinical and instrumental assessments of sleep parameters	Improvement in sleep parameters, specifically the duration of continuous sleep and number of wakings at night (no *P* values given)
Ständer et al [21] (2016)	AD	Sertaconazole 2% cream	1	RCT	70	2% cream BID for 4 wk, followed by a 2-wk washout period	SCORAD	No significant difference was observed between experimental and test groups (*P* values not given)
Kubota et al [22] (2009)	AD	Corticosteroids and tacrolimus (both topical)	2	Open label, uncontrolled	28 (pediatric)	Phase 1: 0.03% tacrolimus ointment QDam^u^ and weak or strong potency corticosteroid ointment QDpm for 2 wk Phase 2: 0.03% tacrolimus ointment BID on weekdays with concurrent corticosteroids on weekends for 2 wk Phase 3: 0.03% tacrolimus ointment BID for 2 wk Phase 4: emollient QD with 0.03% tacrolimus as necessary for 6 weeks	4-point sleep disturbance scale	Decrease from baseline of 1 to 0.03 and 0.04 at 6 and 12 wk, respectively (*P*<.001)
Doss et al [23] (2009)	AD	Tacrolimus 0.03% ointment	1	RCT	240	Tacrolimus 0.03% ointment BID until clearance, maximum 3 wk, then if lesions still present, QD for another 3 wk	Sleep quality (1-100 mm VAS)	Quality of sleep improved in both tacrolimus and flutacisone treatment groups, with no significant between-group difference (n=236, –91.5% vs n=237, –92.6%)
Leo et al [24] (2004)	AD	Pimecrolimus cream 1%	2	RCT	19	Pimecrolimus cream 1% and control cream applied BID for 2 wk	Actigraph motion logger was used to assess periods of sleep and wakefulness	Sleep parameters generated from the actigraphy were not significantly different between the 2 groups
Jaworek et al [25] (2020)	AD	UV-B/cyclosporine	2	Open label, uncontrolled	42	UV-B: 0.22-0.26 J/cm2 to start, increased until erythema appeared. 3×/wk over 12 wk Cyclosporine: 3.5 mg/kg/d given over 2 doses, then increased after 2 wk. Treatment over 12 wk	AIS^v^	UV-B: reduction from 13.5 (SD 2.4) to 4 (SD 1.1) (*P*<.001) Cyclosporine: reduction from 13.9 (SD 1.8) to 5.9 (SD 0.9; (*P*<.001)
Pustišek et al [26] (2016)	AD	Short-term structured educational program	1	RCT	134	Structured education about AD via a 2-h lecture by a physician specialist and written material	SCORAD and PO^w^ SCORAD index, changes in symptom scores for pruritus and sleep disturbance	Treatment group had significantly lower SCORAD (*P*<.001), PO SCORAD (*P*<.001) index, and sleep disturbance (*P*=.001) at the second visit (2 mo)
Bae et al [27] (2012)	AD	PMR^x^	2	RCT	25	PMR therapy BID for 4 wk	VAS was used for the subjective assessment of LOS^y^	Degree loss of sleep was significantly decreased in the PMR group (*P*=.007)
Deleuran et al [28] (2020)	Pruritus and xerosis	ADE-G1^z^ emollient	2	Open label	5910	BID for 7 d	SCORAD	Reduction from 3.3 (SD 2.9) to 1.3 (SD 1.8) after 7 d (*P*<.001)
Rossi et al [29] (2016)	Pruritus and xerosis	Polidocanol (2%) and prucidine-4 (0.5%) lotion	2	Open label	55	At least BID for 28 d	Insomnia score (0-10)	Reduction from 2.2 to 0 after 28 d (*P*<.001)
Chiricozzi et al [30] (2020)	Prurigo nodularis	Dupilumab	2	Retrospective cohort	27	Single subcutaneous injection of 600 mg, then 300 mg Q2W	Sleep-NRS	Reduction from 8.2 to 1.7 after 16 wk (*P*<.001)
Kontochristopoulos et al [31] (2016)	Psoriasis vulgaris	Calcipotriol-betamethasone dipropionate gel	2	Prospective open-label study	394	Calcipotriol-betamethasone dipropionate gel QD	Subjective Surveys about sleep (1-10 rating)	Significantly lower mean scores for pruritus and sleep disorders (*P*<.001)
Zuberbier et al [32] (2009)	Chronic idiopathic pruritus	Bilastine or levocetirizine	1	RCT	525	Bilastine: 20 mg QD for 28 d Levocetirizine: 5 mg QD for 28 d	5-point sleep disturbance scale	A greater percentage of patients at 14 and 28 d treated with bilastine (n=89, 54.6% and n=106, 62.7%) and levocetirizine (n=103, 66.9%, and n=115, 72.3%) reported no sleep disturbance compared to placebo (n=55, 33.4% and n=76, 42.7%; *P*<.001)

^a^AD: atopic dermatitis.

^b^Q2W: once every 2 weeks.

^c^NRS: numerical rating scale.

^d^RCT: randomized controlled trial.

^e^QW: once weekly.

^f^SCORAD: Scoring Atopic Dermatitis.

^g^POEM: Patient-Oriented Eczema Measure.

^h^VAS: visual analog scale.

^i^PROMIS: Patient Reported Outcome Measurement Information System.

^j^QD: once a day.

^k^Using a linear mixed-effects model after controlling for age and sex.

^l^SASSAD: 6 area, six sign atopic dermatitis.

^m^NTIS: Night Time Itch Scale.

^n^ADSS: Atopic Dermatitis Sleep Scale.

^o^BSA: body surface area.

^p^TC: tandospirone citrate.

^q^QDpm: once a day in the evening.

^r^BID: twice a day.

^s^TST: total scratching time.

^t^EEG: electroencephalogram.

^u^QDam: once a day in the morning.

^v^AIS: Athens Insomnia Scale.

^w^PO: patient oriented.

^x^PMR: progressive muscle relaxation.

^y^LOS: loss of sleep.

^z^ADE-G1: *Aquaphilus dolomiae* extract.

**Table 2:** Adverse effects of treatment.

Disease and therapy			Recommended dosage or treatment schedule		Adverse effects		References
**AD^a^**							
	Dupilumab	300 mg subcutaneous injection Q2W^b^ for 12 wk		Exacerbation of AD symptoms, nasopharyngitis, headache, conjunctivitis, and fatigue		[7-11]	
	Melatonin	3 mg QD^c^ for 4 wk		None		[12]	
	Cyclosporine	Maximum 5 mg/kg QD		Rhinitis, infected eczema, bronchospasm, upper respiratory tract infection, and headache		[13]	
	Tandospirone citrate	30 mg QD for 4 wk		Not mentioned		[16]	
	Abrocitinib	200 mg or 100 mg QD for 12 wk		Not mentioned		[14]	
	Baricitinib	1 mg or 2 mg QD for 16 wk		Not mentioned		[15]	
	Chlorpheniramine	2.5 mL or 5.0 mL QDpm^d^ depending on age		Mentioned but not described		[17]	
	Nitrazepam	5 mg QD for 2 nights, then 5 mg BID^e^ for 1 night, followed by a 4-d washout period		Not mentioned		[18]	
	Trimeprazine	20 mg taken 1 h before sleeping for 3 nights		Not mentioned		[19]	
	Trimipramine	50 mg taken 1 h before sleeping for 3 nights		Not mentioned		[19]	
	Sertaconazole 2% cream	2% cream BID for 4 wk, followed by a 2-wk washout period		Exacerbation of AD		[33]	
	Tacrolimus 0.03% or corticosteroids	0.03% tacrolimus ointment QDam^f^ and topical corticosteroid QDpm for 2 wk Then, 0.03% tacrolimus ointment BID, with addition of topical corticosteroid BID on weekends, for 2 wk Then, topical emollient QD and 0.03% tacrolimus PRN^g^		Folliculitis		[21]	
	Tacrolimus 0.03%	0.03% cream BID until clearance for a maximum of 3 wk Then, if lesions are still present, apply QD for another 3 wk		Burning sensation, pruritus, bronchitis, rhinitis		[22]	
	Pimecrolimus 1%	1% cream BID for 2 wk		Not mentioned		[24]	
	Colloidal oatmeal cream	Apply topically for 2 wk		Not mentioned		[20]	
	UV-B	0.22-0.26 J/cm2 to start, increased until erythema appeared, 3× per wk for 12 wk		None		[25]	
	PMR^h^ therapy	PMR therapy BID for 4 wk		Not mentioned		[27]	
	Structured education program	Single 2-h lecture and written materials		Not mentioned		[26]	
**Pruritus and xerosis**							
	ADE-GI^i^ emollient	BID for 7 d		Skin irritation, itch, and burning sensation		[28]	
	Polidocanol (2%) and prucidine-4 (0.5%) lotion	At least BID for 28 d		Not mentioned		[29]	
**Chronic idiopathic urticarial**							
	Bilastine	20 mg QD for 28 d		Headache and somnolence		[32]	
	Levocetirizine	5 mg QD for 28 d		Headache and somnolence		[32]	
**Prurigo nodularis**							
	Dupilumab	Single subcutaneous injection of 600 mg, then 300 mg Q2W		Conjunctivitis		[30]	

^a^AD: atopic dermatitis.

^b^Q2W: once every 2 weeks.

^c^QD: once a day.

^d^QDpm: once a day in the evening.

^e^BID: twice a day.

^f^QDam: once a day in the morning.

^g^PRN: as needed.

^h^PMR: progressive muscle relaxation.

^i^ADE-GI: *Aquaphilus dolomiae* extract.

**Table 3:** Funding sources.

Study	Disease	Funding sources
Fargnoli et al [7] (2019)	AD^a^	None
Cork et al [8] (2019)	AD	Sanofi/Regeneron
Tsianakas et al [9] (2017)	AD	Sanofi/Regeneron
Tofte et al [10] (2018)	AD	Sanofi/Regeneron
Paller [11] (ongoing clinical trial)	AD	Not mentioned
Chang et al [12] (2016)	AD	National Taiwan University Hospital and the Yonghe Cardinal Tien Hospital
Harper et al [13] (2000)	AD	Novartis Pharmaceuticals
Silverberg et al [14] (2021)	AD	Pfizer
Lio et al [15] (2021)	AD	Eli Lilly and Company
Kawana et al [16] (2010)	AD	Not mentioned
Munday et al [17] (2002)	AD	Not mentioned
Ebata et al [18] (1997)	AD	Not mentioned
Savin et al [19] (1979)	AD	May and Baker Ltd
Parikh-Das et al [20] (2017)	AD	Johnson & Johnson
Ständer et al [21] (2016)	AD	Not mentioned
Kubota et al [22] (2009)	AD	None
Doss et al [23] (2009)	AD	Astellas Pharma Europe Limited
Leo et al [24] (2004)	AD	Not mentioned
Jaworek et al [25] (2020)	AD	Not mentioned
Pustišek et al [26] (2016)	AD	None
Bae et al [27] (2012)	AD	Korea Health 21 R&D Project
Deleuran et al [28] (2020)	Pruritus and xerosis	Pierre Fabre Dermo-Cosmetique
Rossi et al [29] (2016)	Pruritus and xerosis	Not mentioned
Chiricozzi et al [30] (2020)	Prurigo nodularis	None
Kontochristopoulos et al [31] (2016)	Psoriasis vulgaris	Leo Greece
Zuberbier et al [32] (2009)	Chronic idiopathic pruritus	Not mentioned

^a^AD: atopic dermatitis.

#### Systemic

Dupilumab, a monoclonal antibody that exerts its effect by blocking interleukin (IL)-4 and IL-13 signaling, is a frequently studied treatment of AD. Using pooled data from 2 RCTs comprising a total of 1379 patients, 1 study using dupilumab found improvements in 2 sleep measures, Scoring Atopic Dermatitis (SCORAD) and Patient-Oriented Eczema Measure (POEM), in 2 treatment groups compared with a control. SCORAD and POEM are clinical tools used to define the eczema severity and monitor its progression [34]. The 2 cohorts either received a once weekly or once every 2 weeks injection of 300 mg dupilumab. Mean decreases in SCORAD were 3.4 (SE 0.14) and 3.3 (SE 0.14) for the weekly and biweekly treatments, respectively. Both significantly outperformed the placebo group (*P*<.001). In the POEM measure, more patients reported an absence of sleep disturbance in the weekly (n=199, 43.5%) and biweekly (n=234, 51.2%) groups compared with the placebo group (n=81, 17.6%; *P*<.001) [8].

Another study found significant improvement in visual analog scale (VAS) sleep loss scores for patients with AD undergoing dupilumab treatment. In the 2 pooled RCTs, 300 mg injections were given subcutaneously either weekly or every other week. At week 12, the mean decrease in VAS sleep loss scores for weekly injections was 75% (SD 5.13%) and for biweekly was 52% (SD 10.79%) when compared to the 19.7% (SD 6.17%) and 3.8% (SD 11.55%) reductions for each respective placebo group (*P*=.001) [10]. Similarly, another study that investigated 300 mg dupilumab implemented SCORAD VAS scores to assess sleep loss. A mean percentage decrease of 66% (SD 17.78%) was observed in the dupilumab group when compared to the placebo group [9]. In a retrospective cohort study, 109 patients received an initial injection of 600 mg dupilumab followed by another 300 mg every other week. A reduction in the Sleep numerical rating scale was seen in the treatment group at 4 weeks (7 to 3) and at 16 weeks (3 to 2; *P*<.001) [7].

A single ongoing, phase 4 clinical trial using dupilumab for patients with pediatric AD was also found. An estimated 40 participants will be recruited, and a once-weekly subcutaneous injection, based on body weight, will be administered for a total of 12 weeks. Primary sleep outcome measurements include the PROMIS (Patient Reported Outcome Measurement Information System) parent-proxy score, the PROMIS patient score, and a polysomnography-based wake after sleep onset [11].

Melatonin supplementation, given its suppressive effects of autotaxin, has also been attempted as a treatment for sleep disturbance in children with AD [35]. In a double-blinded RCT of 73 children and adolescents, 3 mg of melatonin daily was compared to a placebo. Sleep measures included SCORAD, actigraphy, and subjective assessment. Compared with placebo, melatonin reduced the SCORAD measure by 9.1 (*P*<.001) and reduced sleep-onset latency by 21.4 minutes (*P*=.02) [12].

Cyclosporine therapy, given it reduces epidural nerve density, is another potential treatment option for childhood AD [36]. One prospective randomized, parallel study compared short course versus continuous treatment schedules. A total of 40 pediatric patients either underwent multiple 12-week courses or a continued 1-year course of 5 mg/kg/day cyclosporine. Improvement in sleep disturbance was observed in all treatment groups, although no values or *P* values were given. A tolerability of 80% was observed at week 12 and at the end of the study [13].

Tandospirone citrate, a 5-HT1A receptor agonist, has been used to reduce stress-related symptoms of AD. One RCT of 37 patients assessed the efficacy of tandospirone citrate at a dosage of 10 mg 3 times daily for 4 weeks. Both SCORAD and an insomnia VAS were used to measure changes in sleep. A significant decrease was observed in SCORAD for the treatment group after 4 weeks (*P*<.001), which was not observed with the control group. However, no significant difference was observed between the groups in either SCORAD or the insomnia VAS [16].

Abrocitinib, a JAK1 inhibitor, was assessed in 1 study using pooled data from 3 RCTs, with a total of 942 patients [37]. Patients received 200 mg, 100 mg, or placebo once daily for 12 weeks, with sleep outcomes measured using the Night Time Itch Scale (NTIS). The percentage of patients reporting a change in NTIS score of >4 was 57% (n=207), 42.7% (n=158), and 12.7% (n=27) for 200 mg, 100 mg, and placebo, respectively. Moreover, the percentage of patients who reported a response in NTIS score after 12 weeks, when compared to placebo, was 44.6% for 200 mg (n=162; *P*<.001) and 29.8% for 100 mg (n=110; *P*<.001) [14].

Baricitinib, another JAK inhibitor, was studied in post hoc analysis of data from a phase 3, multicenter, double-blinded RCT of 440 patients, which compared once-daily dosages of 1 mg and 2 mg baricitinib to placebo after 16 weeks. In patients with a baseline body surface area involvement of 10% to 50%, a decrease of at least 1.5 points in the number of nighttime awakenings caused by itch was observed for 2 mg (*P*=.001) but not 1 mg (*P*=.15) groups versus placebo. For patients with a baseline Atopic Dermatitis Sleep Scale score ≥1.5, a decrease of at least 1.5 points in the number of nighttime awakenings caused by itch was observed for both 1 mg (*P*=.04) and 2 mg (*P*<.001) versus placebo. Adverse effects were not mentioned [15].

Chlorpheniramine, an antihistamine, was assessed in 1 RCT of 151 patients with AD, with no significant difference being observed in AD symptoms when compared to placebo. Dosages included 2.5 mL versus 5 mL, given once at night. There was also no difference in the severity of daytime drowsiness and the number of episodes of sleeplessness [17].

Ebata et al [18] investigated nitrazepam, a benzodiazepine believed to reduce itch by sedation, and found no significant effects on nocturnal scratching behavior. Ten patients either took 5 mg nitrazepam once nightly for 2 consecutive days or 10 mg for 1 night, followed by a 4-day washout period. A decrease in frequency in nocturnal scratching bouts was observed at the 10 mg dosage; however, this coincided with an increase in mean scratching duration. Hence, no significant difference was observed in TST% when compared to placebo [18].

Trimeprazine, an antihistamine and sedative, and trimipramine, a tricyclic antidepressant, were tested against a placebo in 1 double-blinded randomized trial of 12 patients with severe AD. Patients underwent a 3-day treatment course of either 20 mg trimeprazine tartrate, 50 mg trimipramine maleate, or placebo once at night. Electroencephalogram recordings were used to measure wakefulness and stages of sleep. No significant differences were found in total time asleep, sleep latency, or wakefulness once asleep [19].

#### Topical

Sertaconazole, an antifungal that inhibits ergosterol synthesis, was studied in 1 double-blind RCT with 70 subjects [33]. Topical 2% sertaconazole cream was applied twice daily for 4 weeks. The SCORAD measure was used to assess changes in sleep. No significant differences were observed between sertaconazole and the vehicle control [21].

Given AD has been hypothesized to arise from immune dysregulation, studies have also examined tacrolimus, a calcineurin inhibitor that inhibits T-cell proliferation [38]. In 1 study, 28 patients were treated with sequential application of topical tacrolimus and corticosteroids in a 4-phase treatment process. In phase 1, patients applied 0.03% tacrolimus ointment every morning and a corticosteroid ointment every night for 2 weeks. In phase 2, the same concentration of tacrolimus ointment was applied twice daily for 2 weeks, with the addition of the corticosteroid ointment on weekends. Phase 3 eliminated the corticosteroid treatment while continuing tacrolimus twice daily for 2 weeks. The fourth and final phase involved the application of an emollient once daily, with tacrolimus application as needed for a total of 6 weeks. A 4-point sleep disturbance scale was used to assess changes in sleep disturbance. A decrease was observed from a baseline of 1.0 to 0.03 and 0.04 at 6 and 12 weeks, respectively (*P*<.001) [22].

Topical tacrolimus has also been compared to topical fluticasone, a corticosteroid agent and T-cell proliferation inhibitor [39]. Tacrolimus or fluticasone was applied twice daily until the clearance of lesions. If lesions persisted by week 3, the application was continued once daily for an additional 3 weeks. Sleep quality, measured with VAS, improved in both groups but with no significant difference [23].

Another immunomodulatory agent, which inhibits calcineurin, pimecrolimus, has also been tested for AD treatment [40]. In 1 RCT of 19 pediatric patients experiencing AD, pimecrolimus cream was not shown to be effective in improving sleep parameters, specifically sleep duration and wakefulness. Moreover, no difference was observed in sleep parameters between the treatment and control groups [24].

Colloidal oatmeal, an anti-inflammatory and antihistaminergic agent via avenanthramide, is another potential treatment option for AD [41]. In 1 study, 23 infants and toddlers with AD received colloidal oatmeal lotion for 14 days. Sleep quality was assessed “clinically and instrumentally,” with improvement noted in continuous sleep duration and number of wakings. The degree and timing of these improvements were not specified [20].

#### Procedural

UV phototherapy is a common treatment for dermatoses by stimulating prostaglandins and cytokine synthesis [42]. One study compared the efficacy of UV-B and cyclosporine on sleep disturbance secondary to AD, by implementing the Athens Insomnia Scale. UV-B dosage began at 0.22-0.26 J/cm^2^ and was increased every second session until erythema appeared. Doses were given 3 times a week, with a maximum dose of 0.56 J/cm^2^. The baseline cyclosporine dosage was 3.5 mg/kg/d, divided in 2 doses, and increased after 2 weeks, with a maximum dose of 5 mg/kg/d. Patients either received UV-B or cyclosporine for a period of 12 weeks. Itch reduction was achieved in both treatment groups, with more significant itch reduction in the UV-B cohort (*P*<.001). Moreover, UV-B treatment resulted in a reduction from 13.5 to 4 in mean Athens Insomnia Scale scores (*P*<.001). Cyclosporine, likewise, led to a mean reduction of 13.9 (SD 1.8) to 5.9 (SD 0.9; *P*<.001) [25].

Progressive muscle relaxation (PMR) therapy is a form of psychological therapy primarily used for stress management [43]. In 1 RCT, 25 patients were randomly assigned to either receive 1 month of PMR therapy in addition to conventional treatment or only undergo 1 month of conventional treatment. A significant decrease in sleep loss horizontal VAS scores was observed in the PMR group but not the control group (*P*=.007) [27].

Structured parental intervention, which consisted of a 2-hour physician lecture, was evaluated for improving sleep in 1 study of 134 children with AD. Sleep parameters were measured using SCORAD, patient-oriented (PO) SCORAD index, and sleeplessness and evaluated at the second visit (2 mo after initial evaluation). By the second visit, the mean SCORAD scores for the intervention group were 23.08 (SD 15.188) versus 36.44 (SD 16.760) for the control group (*P*=.001), and PO SCORAD index scores were 24.92 (SD 16.572) and 38.31 (SD 16.253), respectively (*P*=.001). Likewise, sleep disturbance means were 2.94 (SD 2.981) for the intervention group and 4.69 (SD 2.943) for the control group (*P*=.001) [26].

### Treatment for Pruritus and Xerosis

*Aquaphilus dolomiae* extract, a biomanufactured derivative from *Aquaphilus dolomiae*, has been shown in vitro to counteract the mitogenic effects on CD-4^+^ T-cells, which may result in immunosuppressive effects [44-46]. One open-label study of 5910 patients evaluated the effects of an emollient containing *Aquaphilus dolomiae* extract on pruritus and xerosis severity and sleep disturbance. The application was done twice daily for 7 days. A VAS derived from the SCORAD index was used to measure changes in sleep, with 0 representing “no sleep disturbance” and 10 representing “very severe sleep disturbance.” After the treatment period, a decrease from 3.3 to 1.3 in sleep score was observed (*P*<.001) [28].

Polidocanol is a local anesthetic agent shown to have an antipruritic effect on nonhistamine-induced itch [47]. Prucidine-4 is an antipruritic formulation reported to act as a TRPV1 antagonist [48]. One open-label study with 55 patients with xerosis compared compounded polidocanol (2%) to prucidine-4 (0.5%) lotion. Both were applied at least twice daily for 28 days, and each led to a decrease in insomnia scores from 2 to 0 after 28 days (*P*<.001) [29].

### Treatment for Chronic Idiopathic Urticaria

Bilastine and levocetirizine, both H_1_-receptor antagonists, were compared in 1 RCT on sleep parameters of 525 patients diagnosed with chronic idiopathic urticaria. Patients either received 20 mg bilastine or 5 mg levocetirizine once daily for 28 days. Assessment of sleep was measured using a 5-point sleep disturbance scale. Overall, by 14 and 28 days, a greater percentage of patients treated with bilastine (n=89, 54.6%; n=106, 62.7%) and levocetirizine (n=103, 66.9%; n=115, 72.3%) reported having no sleep disturbance, respectively, when compared to placebo (n=55, 33.4%; n=76, 42.7%; *P*<.001) [32].

### Treatment for Prurigo Nodularis

One retrospective study of dupilumab was performed with 27 patients with prurigo nodularis. An initial loading dose of 600 mg was given via subcutaneous injection, followed by 300 mg injections every other week. Mean sleeplessness numerical rating scale scores decreased from a baseline of 8.2 (SD 2.0) to 1.7 (SD not given), after 16 weeks of treatment (*P*<.001) [30].

### Treatment for Psoriasis Vulgaris

One open-label study evaluated the effect of calcipotriol-betamethasone dipropionate gel in 394 Greek patients with psoriasis vulgaris. The gel was applied once daily for 4 weeks, with various quality-of-life assessments performed before and after the treatment period. Sleep disturbance was measured using a subjective survey, ranging from 1 (no itching) to 10 (worst state). By the end of the treatment period, a significant decrease in mean score from baseline was observed (*P*<.001) [31].

## Discussion

### Summary

In this review, a variety of studies assessed treatment efficacy in influencing sleep parameters for dermatological conditions, predominantly AD. Given the inclusion of sleeplessness in SCORAD, a robust clinical tool to assess atopic dermatitis, this result is not surprising. Therefore, the inclusion of a sleep measure into clinical tools assessing other kinds of dermatological conditions may be beneficial.

In terms of atopic dermatitis, 3 broad classes of treatment were found: systemic, topical, and procedural. Among systemic treatments, dupilumab was the most studied. It was shown to be efficacious in improving sleep parameters. All published dupilumab studies for AD found significant improvements in sleep disturbance, sleep quality, and sleep loss. This may be secondary to the notable effect of dupilumab to rapidly reduce pruritis in patients with AD [49]. The adverse effects (AEs) of dupilumab, however, should be taken into consideration (Table 2). While usually mild, AEs were frequently observed in dupilumab treatment studies. Other systemic treatments including melatonin, abrocitinib, and cyclosporine showed promising results in improving sleep parameters, but further studies will be needed to establish their efficacy. While not always discussed in these studies, the potential AEs of these other treatments also warrant further consideration. For example, abrocitinib and baricitinib have been associated with increased risk for infections and malignancy [50,51]. Clinicians should keep such AEs in mind, particularly the immunosuppressive effects of abroctinib, barictinib, and to a lesser extent dupilumab, when attempting a new treatment plan.

The current gap in research on nonimmunosuppressive treatment options for sleep disturbances also deserves mention. Of note, there was a lack of studies for many medications commonly prescribed by dermatologists to assist with sleep, such as sedating antihistamines, tricyclic antidepressants, mirtazapine, gabapentin, and naltrexone. Further research into the sleep-related effects of these drugs is warranted.

Contrary to systemic treatments, topical treatments for AD were mostly ineffective in influencing sleep. One study using a steroid-tacrolimus combination treatment did find a significant improvement in a 4-point sleep disturbance measure in pediatric patients. However, given a relatively small sample size and lack of blinding, more evidence is needed to verify this effect. Similarly, procedural treatments for AD such as UV-B, PMR, and structured educational programs appeared highly efficacious in improving sleep, but replication is needed before definite conclusions can be drawn. The relative absence of AEs in these interventions, however, makes them promising directions for future research.

For dermatoses other than AD, only a limited number of treatment studies were found that included any kind of sleep measure. For the studies that were found, however, significant benefits to sleep measures were observed. Given the importance of sleep for patients’ quality of life, broader inclusion of sleep measures in treatment studies, whether as part of a standardized clinical assessment tool or as a standalone assessment, merits further consideration.

Several limitations of this review exist. First, there was a lack of homogeneity in the studies found, which precluded the performing of a meta-analysis. The time range of our studies also only encompasses studies up to 2021, meaning newer research may not have been included. Additionally, the nature of funding for some of the treatment studies could have introduced a source of bias, especially for recently introduced medications such as dupilumab (Table 3). Lastly, while the methodology of this review fulfilled most requirements of AMSTAR 2 (A Measurement Tool to Assess systematic Reviews), it did not include a risk of bias assessment [52].

### Conclusions

Assessment of sleep changes remains an understudied aspect of dermatological treatment studies. A majority of the studies that did measure changes in sleep were related to AD; however, studies related to pruritus and xerosis, prurigo nodularis, psoriasis vulgaris, and chronic idiopathic pruritus were also found. Dupilumab was most effective at improving sleep measures for AD, but its side effect profile must be taken into consideration. For other dermatoses, given the small number of studies found, further research is needed to establish their efficacy. A broader inclusion of sleep change measures in dermatological treatment studies is warranted.

## Appendix

Multimedia Appendix 1 PRISMA (Preferred Reporting Items for Systematic Reviews and Meta-Analyses) checklist.

## Abbreviations

AD: atopic dermatitis
AE: adverse effect
AMSTAR: A Measurement Tool to Assess systematic Reviews
IL: interleukin
NTIS: Night Time Itch Scale
PMR: progressive muscle relaxation
PO: patient-oriented
POEM: Patient-Oriented Eczema Measure
PROMIS: Patient Reported Outcome Measurement Information System
RCT: randomized controlled trial
SCORAD: Scoring Atopic Dermatitis
SORT: Strength of Recommendation Taxonomy
VAS: visual analog scale
